# Tuning the Surface Wettability of Cyclic Olefin Copolymer by Plasma Treatment and Graphene Oxide Deposition and Reduction

**DOI:** 10.3390/polym13142305

**Published:** 2021-07-14

**Authors:** Fadi Dawaymeh, Yawar Abbas, Maryam Khaleel, Anas Alazzam, Nahla Alamoodi

**Affiliations:** 1 Center of Catalysis and Separation (CeCaS), Department of Chemical Engineering, Khalifa University, Abu Dhabi P.O. Box 127788, United Arab Emirates; 100052916@ku.ac.ae; 2 System on Chip Center (SoCC), Department of Physics, Khalifa University, Abu Dhabi P.O. Box 127788, United Arab Emirates; yawar.abbas@ku.ac.ae; 3 Research and Innovation Center in Carbon Dioxide and Hydrogen (RICH), Center of Catalysis and Separation (CeCaS), Department of Chemical Engineering, Khalifa University, Abu Dhabi P.O. Box 127788, United Arab Emirates; maryam.khaleel@ku.ac.ae; 4 System on Chip Center (SoCC), Department of Mechanical Engineering, Khalifa University, Abu Dhabi P.O. Box 127788, United Arab Emirates

**Keywords:** surface wettability, graphene oxide, plasma treatment, cyclic olefin copolymer, GO reduction

## Abstract

Selective altering of surface wettability in microfluidic channels provides a suitable platform for a large range of processes, such as the phase separation of multiphase systems, synthesis of reaction controlled, nanoliter sized droplet reactors, and catalyst impregnation. Herein we study the feasibility to tune the wettability of a flexible cyclic olefin copolymer (COC). Two methods were considered for enhancing the surface hydrophilicity. The first is argon/oxygen plasma treatment, where the effect of treatment duration on water contact angle and COC surface morphology and chemistry were investigated, and the second is coating COC with GO dispersions of different concentrations. For enhancing the hydrophobicity of GO-coated COC surfaces, three reduction methods were considered: chemical reduction by Hydroiodic acid (HI), thermal reduction, and photo reduction by exposure of GO-coated COC to UV light. The results show that as the GO concentration and plasma treatment duration increased, a significant decrease in contact angle was observed, which confirmed the ability to enhance the wettability of the COC surface. The increase in hydrophilicity during plasma treatment was associated with the increase in surface roughness on the treated surfaces, while the increase during GO coating was associated with introducing oxygen-containing groups on the GO-coated COC surfaces. The results also show that the different reduction methods considered can increase the contact angle and improve the hydrophobicity of a GO-coated COC surface. It was found that the significant improvement in hydrophobicity was related to the reduction of oxygen-containing groups on the GO-coated COC modified surface.

## 1. Introduction

Several materials are used in the fabrication of microfluidic devices. Recently, the fabrication of these devices has relied on polymer based materials such as polydimethylsiloxane (PDMS) and cyclic olefin copolymer (COC) [1,2,3,4]. This attention is related to their mechanical and chemical characteristics that make the fabrication of devices faster, easier, and cheaper in comparison to other materials such as silicon and glass [1,2]. Furthermore, PDMS and COC have other desirable features, such as being biologically inert, optically transparent, nonflammable, low cost, and nontoxic [2]. Therefore, it becomes an alternative material for different micromanufacturing models. However, PDMS and COC are hydrophobic in nature, which makes them unsuitable for many bio-applications [5,6]. Several physical and chemical techniques have been developed to modify the surface wettability of polymer based microchannels for selective applications. These include plasma treatment, chemical vapor deposition (CVD), graft polymer coating, the sol-gel technique, and layer by layer (LBL) deposition [2,7,8,9,10]. The oxidation of a PDMS polymer surface by oxygen plasma suffers from being temporary [11]. Storing the oxidized surfaces in water and other high surface-energy media can be used to preserve the hydrophilic surface for some time before the polymer reverts to its original surface properties. However, the exposure of the surface to air or other low surface-energy media results in its hydrophobic recovery [12]. On the other hand, chemical vapor deposition can produce long lasting coatings but only has an effect on chips that are not assembled or connected to other parts or accessories [13]. To achieve a high coating effectiveness, the vapor used for coating should have unrestricted access to the substrate. In a PDMS assembled channel, the use of graft photopolymerization for covalent surface modification faces some challenges, because it does not react on the wall but in the channel cavity. Hu et al. [14] developed a method to solve this challenge by photoinitiator preadsorption before polymerization. However, in addition to multiple washings, this method also requires the manual injection of a photoinitiator and monomer solution into the chip, which makes the process complicated. The sol-gel technique for PDMS modification was used by Abate et al. [15]. This method permanently modifies the PDMS with a glass coating, which has strong resistance to organic solvents and can customize its performance. However, the coating thickness can sometimes be as high as 10 mm, which affects the size of the channel and interferes with the complex geometry of the device. In addition, the critical gelation reaction time lasts for few seconds, making the process delicate and uncontrollable. LBL is simple, clear, and versatile. However, injecting the solution and removing it during washing and the formation of layers is a manual process.

Graphene oxide (GO) and its reduced form are two of the most interesting materials in recent research due to their superior physical and chemical properties. The unique planar structure and the two dimensionality of graphene oxide enable the development of flexible optoelectronics, transparent electrodes, energy harvesting devices, and photodetectors [16,17,18]. Graphene oxide has a high concentration of oxygen-containing functional groups, which can form hydrogen bonds when in contact with water. Recently, Alazzam and Alamoodi [4,19] have proposed a new surface modification method, taking advantage of the hydrophilicity of GO to pattern water wet surfaces on COC. The COC substrates were patterned with graphene oxide by photolithography. The hydrophilicity of COC and GO-coated COC surfaces was evaluated using water contact angle measurements. The results showed that the contact angle of the COC surface was 120°, indicating strong hydrophobicity, whereas GO-coated COC showed a strong hydrophilic surface with a contact angle of 10°.

While GO is hydrophilic, the oxygen-containing functional groups can be removed by several methods, called reduction methods, to restore graphene like hydrophobicity. These methods include thermal treatment, chemical reducing agents, ultraviolet reduction, electric current, selective laser reduction, or photothermal reduction [16,20,21,22,23,24]. The reduction extent of GO depends on the process conditions of the method used. Although there are numerous studies that investigate the different properties of GO, few have reported on tuning the surface wetting properties of polymers by means of GO coating and reduction process conditions. This is of particular interest in the case of functional coatings for selective patterning of wettability. Such coatings are particularly important for use in biomaterials, self-cleaning surfaces, smart textiles, microelectronics, and microfluidics [25,26,27]. In this work, we study the feasibility to tune the wettability of flexible COC. Two methods were considered for enhancing the surface hydrophilicity. The first is plasma treatment, where the effect of treatment duration on water contact angle and COC surface morphology and chemistry was investigated, and the second is coating COC with GO dispersions of different concentrations. For enhancing the hydrophobicity of GO-coated COC surfaces, three reduction methods were considered: chemical reduction by Hydroiodic acid (HI), thermal reduction, and photo reduction by exposure of GO film to UV light.

## 2. Materials and Methods

### 2.1. Materials

Disc shaped COC polymer substrates (diameter: 6.5 cm, thickness: 175 µm, Tg: 132 °C, MICROFLUIDIC CHIPSHOP, Jena, Germany), aqueous dispersion of GO (4 mg/mL, SIGMA-ALDRICH, Missouri, US), aqueous dispersion of GO (2.5 wt%, GRAPHENEA, San Sebastian, Spain), hydroiodic (HI) acid (≥47.0%, Sigma-Aldrich, MO, USA), ethanol (absolute, >99.8%, Fisher Chemical, Waltham, MA, USA), isopropanol (absolute, >99.8%, Fisher Chemical, Waltham, MA, USA) and deionized water (DI) were used in this study.

### 2.2. Preparation of GO-Coated COC

**Figure 1:**
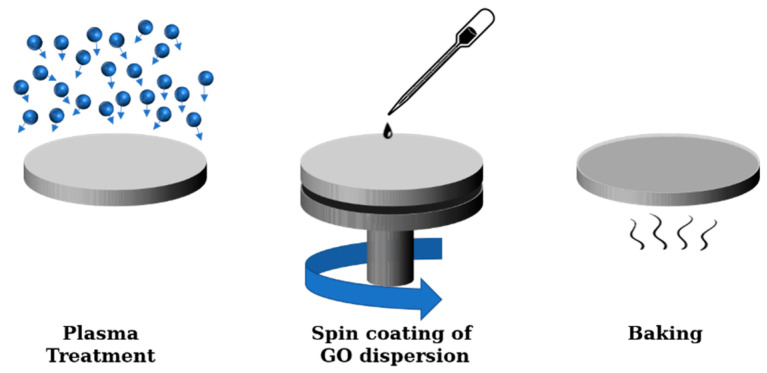
Schematic illustration of the experimental procedure followed to deposit GO films on COC substrates via spin coating.

Graphene oxide dispersions of water and ethanol at a volume ratio of 1:4 were prepared and used to increase the uniformity of the GO layer coated on the COC substrate. GO (4 mg/mL, SIGMA-ALDRICH, St. Louis, MO, USA) dispersions with different concentrations (0.1, 1, 2, 3 and 4 mg/mL) were prepared to study the effect of GO concentrations on the wettability of the COC surface. Dispersions of 4 mg/mL prepared from GO (2.5 wt%, GRAPHENEA, San Sebastián, Spain) were used for all other experiments. The procedure to coat GO on COC substrates is illustrated schematically in Figure 1. First, the COC substrate was cleaned using isopropanol and DI. Next, the substrate was treated with an argon/oxygen low pressure inductive plasma (HARRICK PLASMA, Model: Expanded plasma cleaner/PDC-002, Ithaca, NY, USA) for a fixed duration (5, 20, 60 and 120 min) to increase the surface energy of the substrate and enhance the bonding between GO and COC substrate [19]. Plasma treatment was performed at a pressure of 626 mtorr, gas flowrates of 1–2 scfh, and a maximum power of 29.6 W with radio frequency (RF) of 12 MHz. As will be discussed in the results section, plasma treatment for more than 5 min did not result in noticeable changes in contact angle for GO-coated COC substrates. Therefore, for the remaining experiments, the time for plasma treatment was fixed at 5 min.

After plasma treatment, a few drops of graphene oxide dispersion were dispensed on top of the substrate followed by spin coating at 3000 rpm for 1 min. Finally, the coated substrate was baked at 60 °C for 2 min.

### 2.3. Reduction of GO-Coated COC

#### 2.3.1. UV Reduction

A UV flood exposure system (BACHUR and ASSOCIATES, Model: LS-150-5C2 NUV exposure system, Santa Clara, CA, USA) was used to reduce the GO coating on the COC substrate in a controlled manner. The lamp emits ultraviolet light with wavelengths of 365 nm and 400 nm with an intensity of 10 mW/cm^2^. The lamp was warmed for 20 min before it was used in the experiments. Then, GO-coated COC substrates were placed individually under the UV lamp and treated for different durations (10, 30, 60, 120 and 180 min) under atmospheric pressure.

#### 2.3.2. HI Reduction

The GO-coated COC substrates were immersed in (47.0%) HI acid for different durations (10, 30 and 180 min). After that, the partially reduced GO-coated COC substrates were washed with DI water and dried with compressed air.

#### 2.3.3. Thermal Reduction

The GO-coated COC substrates were heated on a hot plate at different temperatures (80, 100 and 120 °C) for 30 min, and then cooled to room temperature.

### 2.4. Characterization

The topographic images of the plasma treated samples were obtained by using the AC imaging mode of atomic force microscopy (AFM) of ASYLUM MFP-3D. The high frequency silicon tip with apex diameter of 30 nm was used for imaging. The resonance frequency of the tip is about 246 kHz. The images were taken for scan area of 20 µm × 20 µm with an optimized scan frequency of 1 Hz. Field emission scanning electron microscope (FESEM) (JEOL JSM-6710FFEG-SEM) was used to investigate and characterize the coating of graphene oxide sheets on the COC substrates. The FESEM micrographs were taken using the acceleration voltage of 5 kV at the working distance of 5 mm. The GO-coated COC substrates characterized by AFM and SEM were coated with GO dispersion with a concentration of 4 mg/mL. The Bruker Alpha FTIR spectrometer system equipped with diamond attenuated total reflectance (ATR) accessory was used to perform FTIR analysis on all treated samples with a wavenumber ranging from 450–4000 cm^−1^. Five repetitions with twenty four scans with a resolution of 4 cm^−1^ were performed on each sample. Postprocessing data using smooth tool in Origin Pro software was performed to remove noisy data in hydroxyl and carbonyl FTIR spectrums. The wettability of the surface was characterized by contact angle measurements using a goniometer (Ossila, Model:L2004A1, Sheffield, UK), in which a 5 µL droplet of deionized water was deposited on the treated surface. The images were analyzed using Ossila Contact Angle (v3.1) software, which uses the tangent method for contact angle measurements. For each sample, the contact angle was measured at a minimum of ten droplets of the same volume at different locations and the results were averaged. All surface characterizations were carried out immediately after each treatment method.

## 3. Results

### 3.1. Effect of Plasma Exposure Time for COC

**Figure 2:**
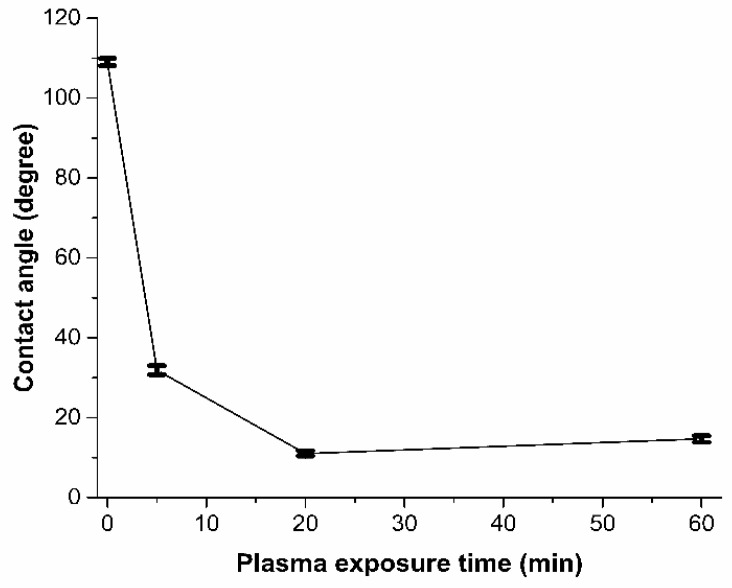
Variation of DI water contact angle with plasma exposure time in plasma treated COC. Each contact angle datapoint shows the average value from ten measurements.

**Figure 3:**
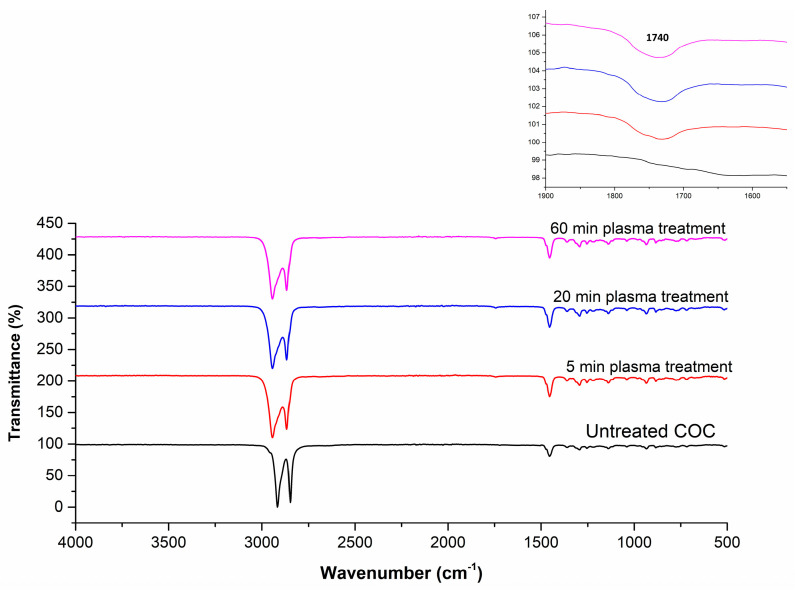
FTIR spectra for plasma−treated COC at different plasma exposure times.

**Figure 4:**
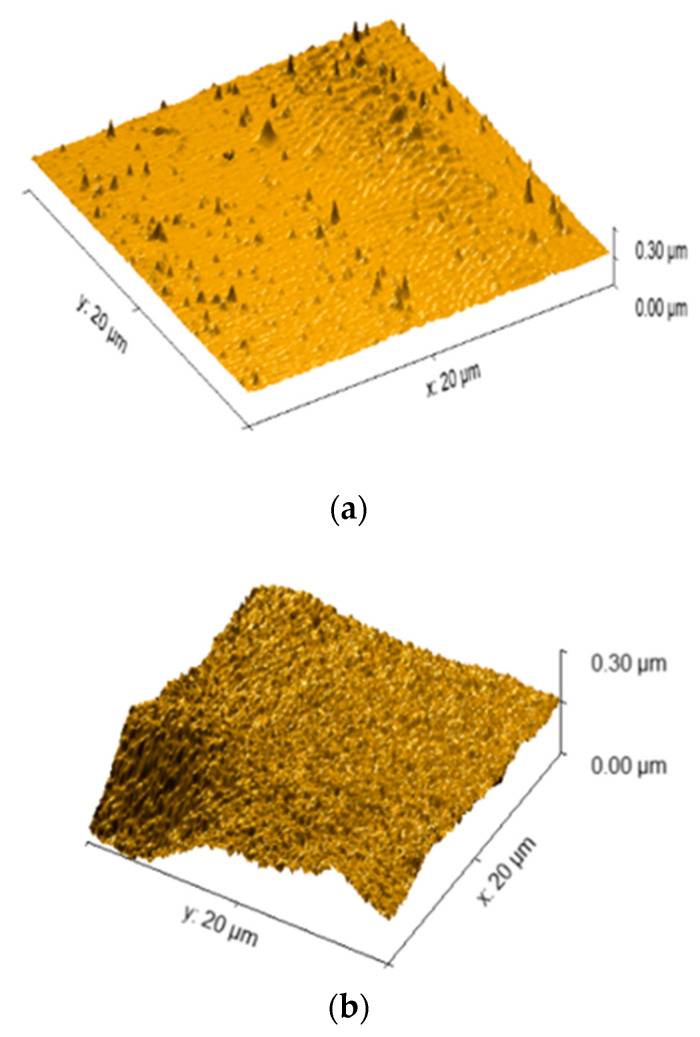

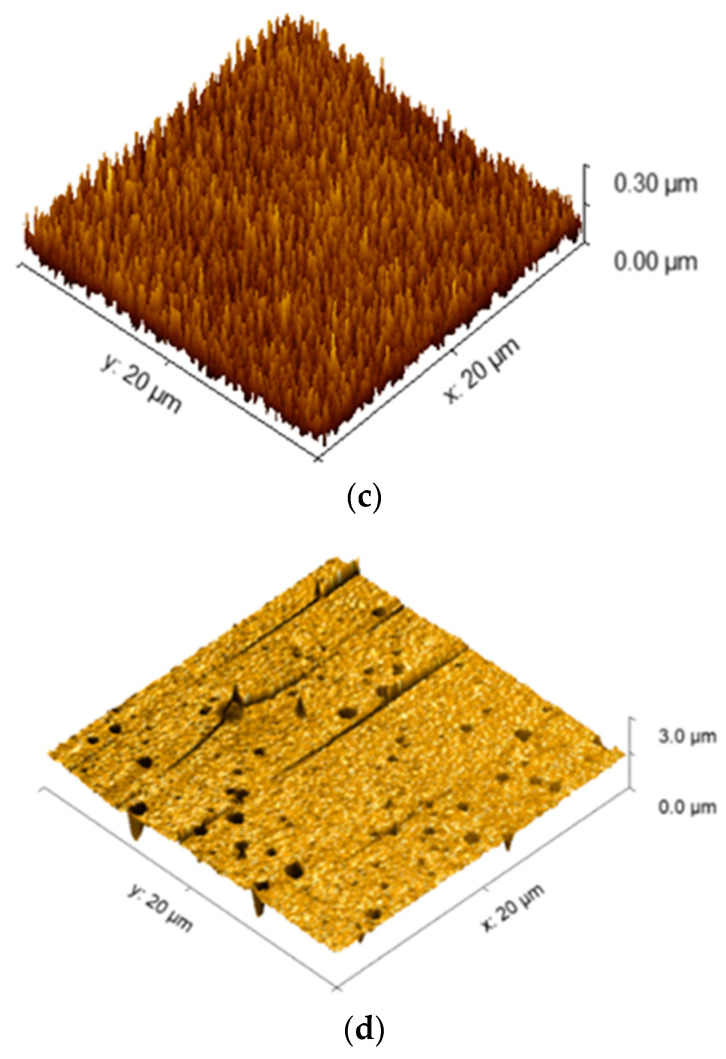
Surface topology of COC substrates using AFM; (**a**) untreated and plasma− treated for (**b**) 5 min, (**c**) 20 min, and (**d**) 120 min.

Plasma exposure causes photo-oxidation, chain session and crosslinking on the COC surface. The active free radicals, ions and electrons generated during plasma treatment induces C-C and C-H bond scission causing shorter polymer chains, the formation of other molecules through recombination reactions and crosslinking on the surface [28]. Several factors affect the outcome of plasma treatment including the power of the plasma unit, the type of plasma (low pressure plasma or atmospheric plasma), and the treatment duration. The use of a high powered plasma treatment or treating polymer surfaces over a long period of time has also been reported to damage polymer surfaces [29]. In the current work, contact angle measurements on the plasma treated COC surfaces were performed directly after plasma treatment. Figure 2 shows the effect of plasma treatment duration on the water contact angle. Untreated COC has a contact angle of 109° while plasma treatment significantly reduces the contact angle to 11° after a 20-min exposure time. It was also observed that for processing times longer than 20 min, the contact angle increased slightly, reaching a value of 15° at 60 min. To understand the contact angle declination, FTIR analysis and AFM imaging were conducted. FTIR analysis was conducted to analyze changes in the functional groups on the COC surface during plasma treatment. The observed FTIR peaks for plasma treated surfaces are shown in Figure 3. It is seen that, generally, there are no major differences between the FTIR results for the plasma treated COC surfaces at different treatment durations. Compared to untreated COC, aldehydes and carbonyl groups are generated during the plasma treatment process (the relevant peak appears at 1740 cm^−1^). These carbonyl-containing groups are formed in the presence of oxygen during plasma treatment [30]. The terminal aldehyde group can be formed by the rearrangement of the peroxy radical intermediate formed by chain scission, or by the decomposition of peroxide and hydroperoxide on the surface of the polymer [28]. AFM images of the surface topography and surface roughness values of untreated and modified COC substrates are shown in Figure 4 and Table 1, respectively. It has been observed that the untreated COC has a smoother surface compared to the plasma modified surface. The surface roughness of the untreated COC substrate was 15.7 nm, while that of the plasma modified COC specimens treated for 5, 20 and 60 min were 33.4 nm, 44 nm and 122.3 nm, respectively. The samples exposed to a long duration of plasma (60 min exposure) showed a very rough surface with occasional holes and cracks observed on the surface (Figure 4d). The increase in the concentration of surface oxygen functional groups and higher surface roughness could contribute to the increase in surface hydrophilicity.

### 3.2. Structural Characterization of COC Coated with GO

**Figure 5:**
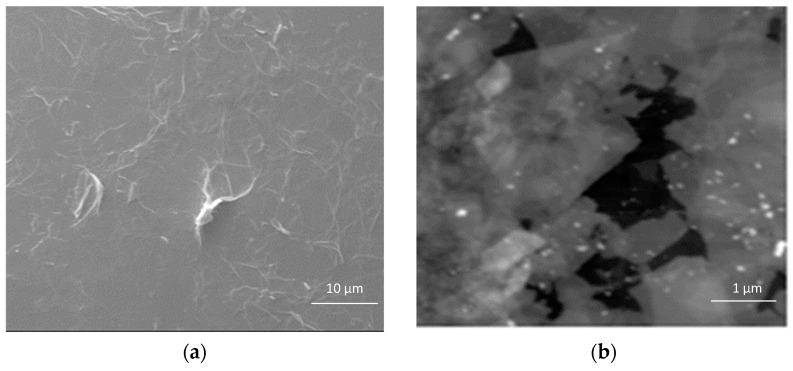
(**a**) SEM and (**b**) AFM images of 4 mg/mL GO−coated COC. The white lines in (**a**) represent the boundaries of the GO flakes.

**Figure 6:**
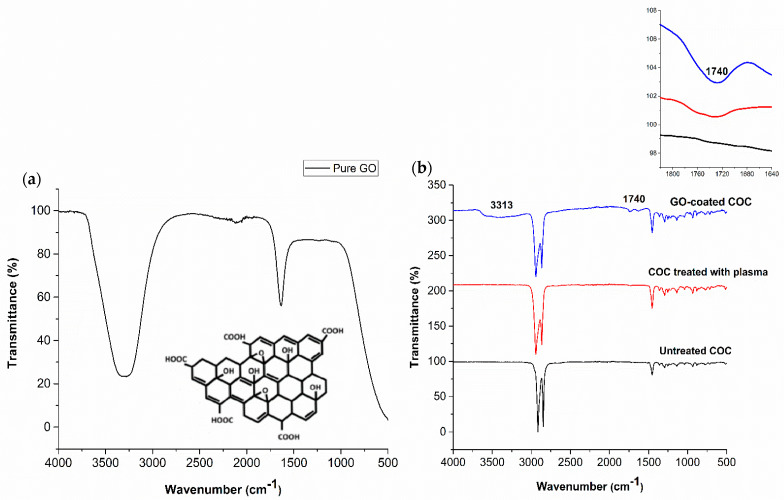
Typical FTIR spectra for (**a**) Graphene oxide and (**b**) untreated COC, COC treated with plasma for 5 min, and 4 mg/mL GO−coated COC.

SEM and AFM images of COC substrate coated with 4 mg/mL GO solution, shown in Figure 5, confirm the uniformity of the GO coating and the interconnection of the sheets, with very little delamination and wrinkles. The uniformity of the GO film on the COC substrate could be a consequence of surface roughness and polar species formed during the plasma treatment process. The FTIR spectra of GO, untreated COC, plasma treated COC, and GO-coated COC, shown in Figure 6, were studied to compare the chemical groups on the COC surface. The FTIR spectra for the untreated COC substrate (Figure 6b), shows a peak at 1452 cm^−1^ for the C-H bending mode and peaks in the range of 2913–2847 cm^−1^ for the stretching mode of C-H (aldehydic H and ethynenic H), in agreement with the literature [28,31,32]. Peaks near 1740 and 3313 cm^−1^ for pure GO and GO-coated COC correspond to C=O and –OH groups, respectively. The absorption peak at 1740 cm^−1^ corresponds to the presence of aldehydes and carbonyl groups generated during plasma treatment, however, the intensity of the peak increases with GO modification, confirming the presence of GO on the COC substrate. The absence of a peak at 3313 cm^−1^ in the plasma-treated COC does not necessarily indicate the absence of hydroxyl groups, which may be present in concentrations below the detection limit of FTIR.

### 3.3. Effect of Plasma Treatment on GO Coating

**Figure 7:**
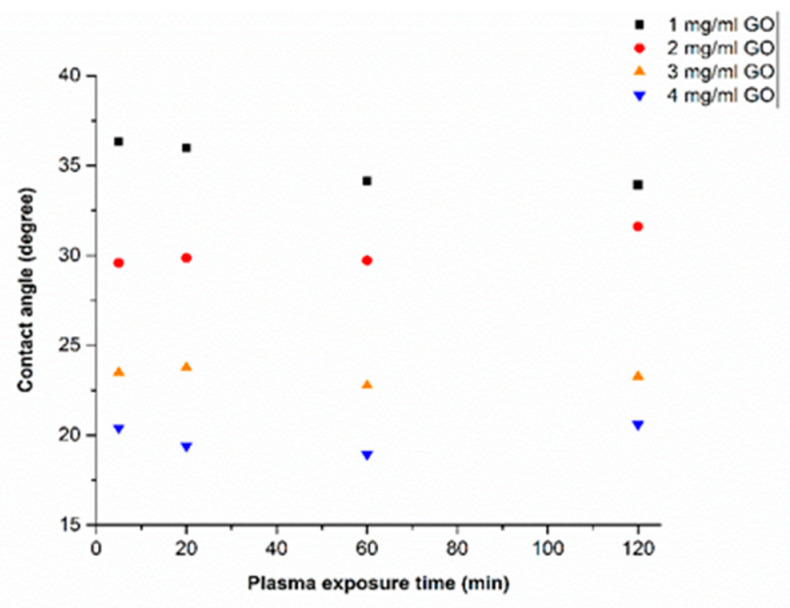
Variation of DI water contact angle with plasma exposure time in plasma pretreated GO−coated COC.

In our recent work [3,4,19] we have shown that treating COC with O_2_/Ar plasma followed by spin coating with GO dispersions resulted in stable GO coatings. Here, we investigated whether there is an apparent effect of plasma treatment on the wettability of the COC after the GO deposition. Contact angle measurements were performed on COC substrates treated with plasma for different durations (5, 20, 60 and 120 min), prior to coating them with different concentrations of GO dispersions (Figure 7). It was observed that the contact angle does not change considerably for each GO concentration when exposed to different plasma treatment durations. Slight changes may result from experimental errors. Therefore, a plasma modification time of 5 min was used in all subsequent experiments.

### 3.4. Effect of GO Concentration

**Figure 8:**
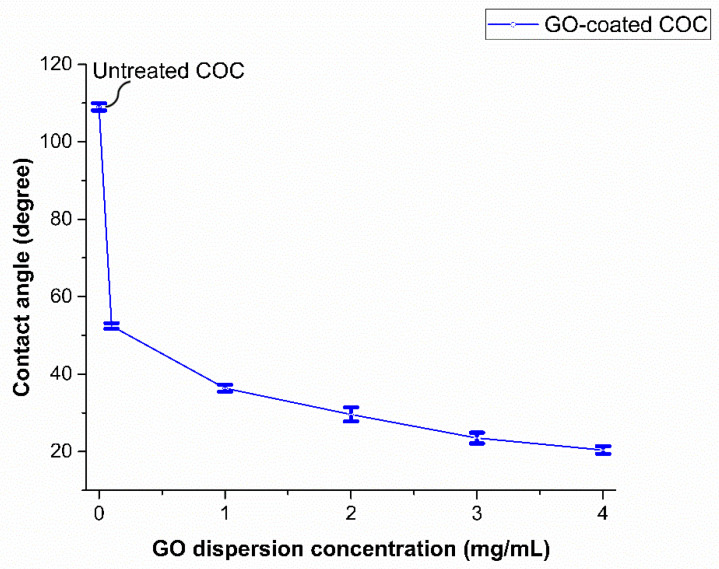
DI water contact angle on GO−coated COC surface as a function of GO dispersion concentration. Contact angle of untreated COC is 109°. All samples were treated for 5 min before the deposition of the GO film.

**Figure 9:**
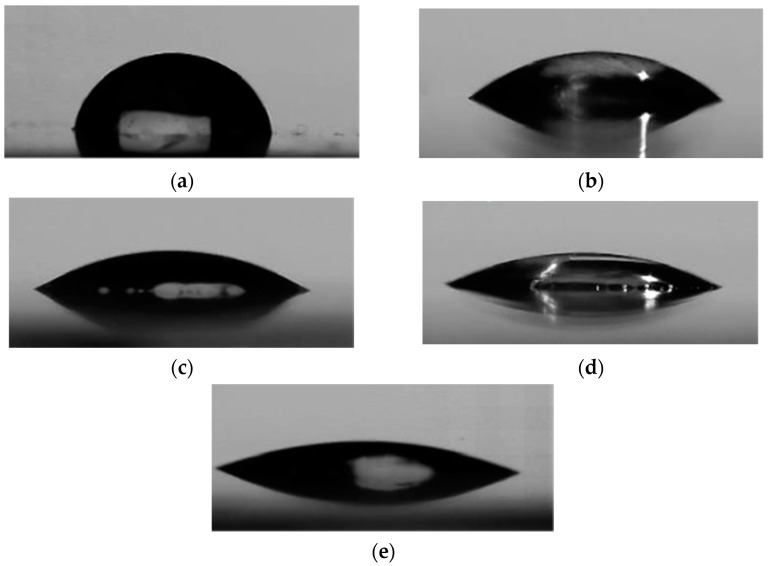
Images of water droplets on the surface of (**a**) COC, and GO−coated COC with GO suspension concentrations of (**b**) 1 mg/mL, (**c**) 2 mg/mL, (**d**) 3 mg/mL, and (**e**) 4 mg/mL at 5 min plasma exposure time. Results were collected immediately after the wafer was fabricated.

**Figure 10:**
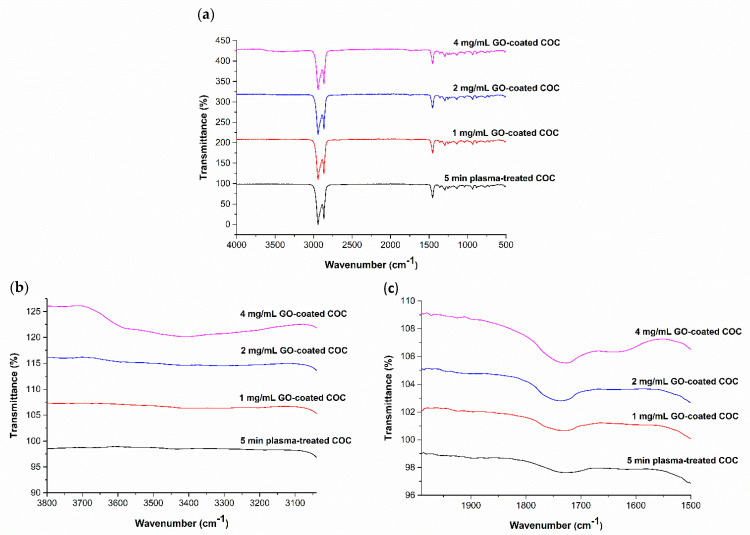
(**a**) FTIR−ATR spectra for five min plasma treated COC and subsequently GO−coated; Expanded regions of FTIR-ATR spectra between (**b**) 3050–3800 cm^−1^ and (**c**) 1500–2000 cm^−1^.

The effect of GO concentration on the wettability of the COC substrate surface was studied using water contact angle measurement. Five GO dispersions were prepared with concentrations of 0.1, 1, 2, 3, and 4 mg/mL, and were deposited on COC substrates pretreated by O_2_/Ar plasma for 5 min using a spin coater at a speed of 3000 rpm. Figure 8 shows the effect of the GO concentration on the contact angle. A decrease in water contact angle with an increase in the concentration of GO dispersion was observed. This behavior can be explained by the fact that graphene oxide is rich in oxygen-containing functional groups (see Figure 6a), which can form hydrogen bonds when in contact with water [33]. Higher concentrations of GO result in the deposition of a larger amount of GO flakes and an increase in the surface concentration of oxygen-containing functional groups, thereby increasing the hydrophilicity of the coated COC. Images of the water droplets on the surface of the GO-coated COC substrates are shown in Figure 9 for GO concentrations up to 4 mg/mL, which resulted in minimum contact angle of 20°. The increase in hydrophilicity with GO concentration can be further confirmed from FTIR analysis. Figure 10 illustrates the functional groups appearing on the COC substrates for different GO concentrations and for plasma treatment of 5 min. It is observed that aldehydes and carbonyl groups are generated during plasma treatment, as discussed in the previous section. They also appear after GO deposition with a more pronounced peak as the GO concentration increases (the relevant peak appears at 1740 cm^−1^). The associated FTIR peak in the range of 3100–3700 cm^−1^ was present in the GO-coated COC samples but not in the plasma treated COC ones. This peak confirms the presence of hydroxyl groups (–OH). The areas and heights of the FTIR peaks are directly proportional to the concentration of each chemical species present on the COC surface [28]. From the changes in peak area and height in Figure 10b,c, it can be seen that the amount of hydroxyl and carbonyl groups increases with the increase of GO concentration, which contributes to the increase in the hydrophilicity of higher concentration GO-coated COC surfaces.

### 3.5. Effect of Ultraviolet (UV), Hydroiodic Acid (HI) and Thermal Treatment on the Wettability of GO-Coated COC

**Figure 11:**
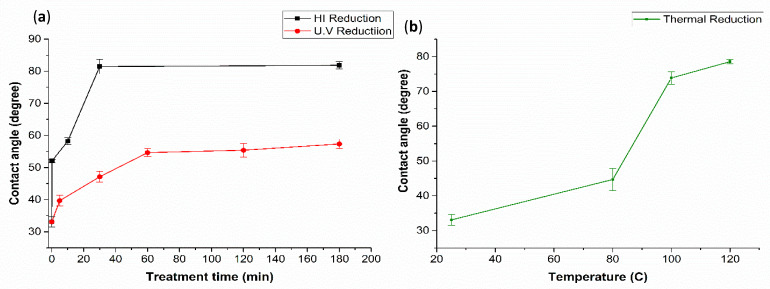
DI water contact angle on the surface of 4 mg/mL GO−coated COC with (**a**) HI and UV reduction time (min) and (**b**) thermal reduction temperature.

**Figure 12:**
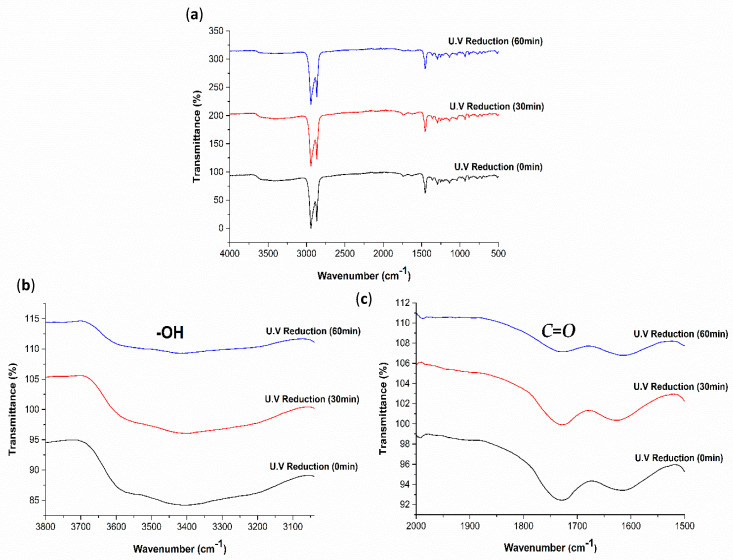
(**a**) FTIR−ATR spectra for 4 mg/mL GO−coated COC treated with UV reduction for different durations and expanded regions of FTIR−ATR spectra between (**b**) 3050–3800 cm^−1^ and (**c**) 1500–2000 cm^−1^.

As seen from the results presented in the previous section, by coating COC substrates with GO dispersions, the wettability of the surface is greatly enhanced, as reflected by the decrease of the contact angle from 109° (untreated COC) to 20° (4 mg/mL GO-coated COC). The hydrophilicity of the GO-coated COC surface can be further tuned by reducing GO using three different approaches: (a) photo reduction using ultraviolet radiation; (b) chemical reduction using hydroiodic acid and (c) thermal reduction by heating the substrate. The effect of photo reduction, chemical (Figure 11a) and thermal reduction (Figure 11b) on the surface hydrophobicity of GO-coated COC surfaces was evaluated by measuring the water contact angle, as shown in Figure 11. The contact angle of the 4 mg/mL GO-coated COC surface was 33° before treatment. Note that this contact angle is higher than the one mentioned in the previous section as this is due to using GO from a different supplier (Graphenea). It can be seen from Figure 11a that the HI acid treatment significantly increases the contact angle to 82° after a 30-minute immersion time. No further change in contact angle was observed for immersion times beyond 30 min. For UV treatment, the contact angle increases slightly with exposure time reaching a maximum value of 55°, with no further change after 60 min (see Figure 11a). This can be attributed to the UV exposure process, where reduction by UV light occurs at the exposed GO flakes at the surface of the coating, this will be further discussed with FTIR spectra in Figure 12. Finally, the thermal treatment of GO-coated COC significantly increases the contact angle reaching 74° at 100 °C. Further increase in temperature, to 120 °C, results in a slight increase in contact angle, to 79° (Figure 11b). This is unlike the HI acid and UV treatment methods, where no further changes in the contact angle were observed after 30 min and 60 min, respectively. It is expected that thermal treatment will increase the contact angle further as the temperature rises. However, this was not investigated because of the COC glass transition temperature of 132 °C. It has been reported that as GO is thermally reduced in the range of 25–130 °C, oxygen-containing groups are mildly removed and the lattice of GO gradually contracts, and at temperatures above 130 °C, oxygen-containing groups disappear significantly and GO lattices contract drastically, resulting in a further increase in the contact angle [34].

**Figure 13:**
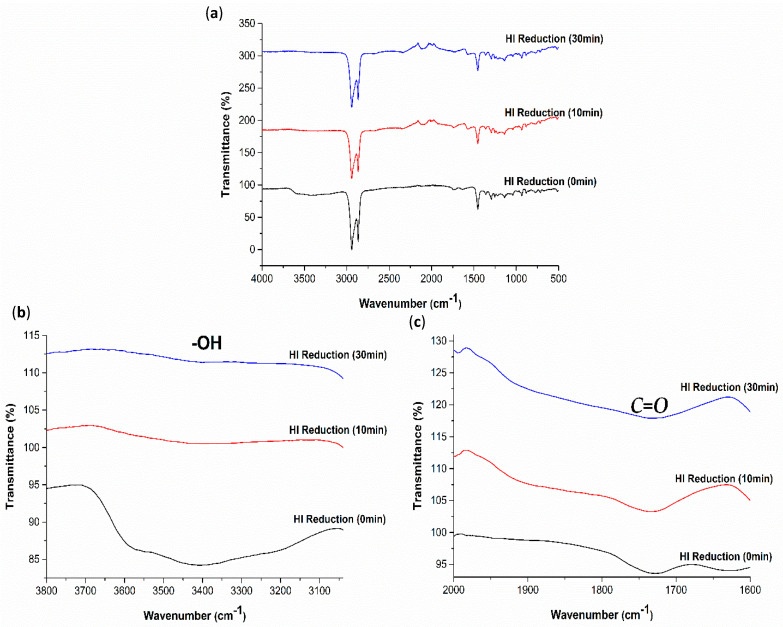
(**a**) FTIR−ATR spectra for 4 mg/mL GO−coated COC treated with HI for different durations and expanded regions of FTIR−ATR spectra between (**b**) 3050–3800 cm^−1^ and (**c**) 1500–2000 cm^−1^.

**Figure 14:**
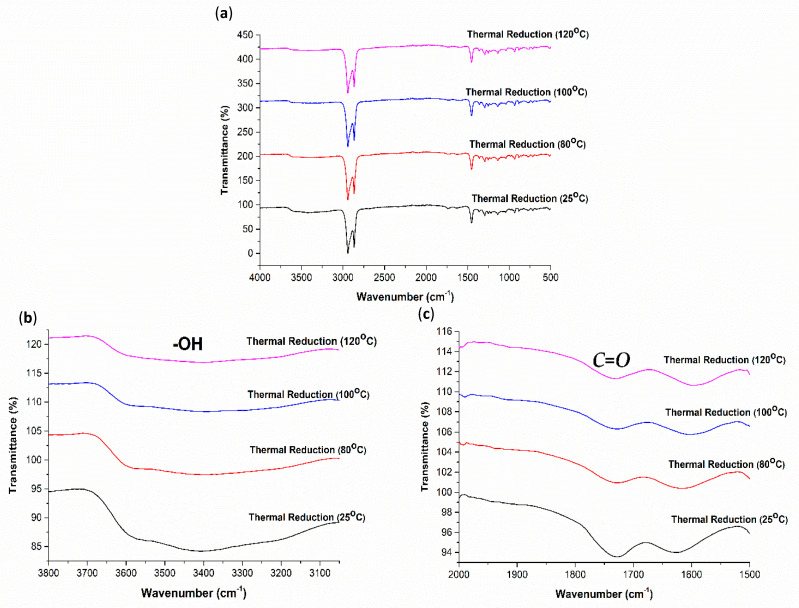
(**a**) FTIR−ATR spectra for 4 mg/mL GO−coated COC treated thermally for different temperatures and expanded regions of FTIR−ATR spectra between (**b**) 3050–3800 cm^−1^ and (**c**) 1500–2000 cm^−1^.

To further evaluate the degree of GO-coated COC reduction by the different methods, the FTIR spectra were obtained, as illustrated in Figure 12, Figure 13 and Figure 14. From the changes in FTIR peak height and area, the amount of hydroxyl and carbonyl groups decrease with the increase in UV exposure time, HI acid immersion time and temperature, which agrees with the reduction of GO and increase of hydrophobicity of GO-coated COC surfaces. The FTIR spectra in Figure 12 shows that after UV exposure of 180 min, hydroxyl groups still exist on the GO coated surface, however, to a lesser extent. The same is observed for the carbonyl groups. In comparison to the FTIR spectra in Figure 13 and Figure 14, the reduction in these groups is minimal with UV exposure. This further confirms that the UV exposure effect is restricted to the exposed GO flakes on the surface of the coating. The expanded FTIR spectra in Figure 13b,c for the HI acid treatments show that the intensity of the oxygen-containing functional groups of the GO-coated COC decreases more than other treatment methods, with the hydroxyl group disappearing completely. This means that the removal of oxygen-containing functional groups in HI acid is greater than other treatment methods for the same duration. At the same time, the absorption peak at 1562 cm^−1^ can be clearly observed during HI reduction, with a higher intensity compared with other methods, which is caused by the stretching vibration of the sp2 carbon–carbon chemical bond in the graphene structure [35,36]. In addition, it has been found that alkyne groups are generated during HI treatment (the relevant peak appears at 2122 cm^−1^). Figure 14 shows a similar trend for the thermal reduction of the GO-coated COC surface, where the oxygen-containing functional groups decrease as the temperature increases. It is worth mentioning that while the contact angle measurements for 30 min treatments in HI acid and at 120 °C are comparable, the FTIR spectra for both cases are different. This suggests that the reduction using HI acid has a greater effect than just reducing the oxygen-containing functional groups, however, further studies are required to confirm this observation.

## 4. Conclusions

The O_2_/Ar plasma treatment and coating of COC with graphene oxide dispersions are effective for enhancing the wettability of natural hydrophobic COC surface. Based on the current work, the hydrophilic characteristic of plasma treated COC is mainly due to its increased surface roughness. Whereas the hydrophilic characteristic of GO-coated COC is mainly due to the increased oxygen-containing groups. Compared with plasma treatment, coating with graphene oxide has better wettability tuning capability because each GO concentration gives a different contact angle measurement as well as selective reduction of GO, specifically chemical and UV exposure reductions, is possible using photolithography techniques, which are difficult to be achieved using plasma treatment. It has been shown that different reduction methods, such as UV exposure, chemical using HI acid, and thermal treatments, can alter the surface wettability from hydrophilic GO-coated COC to hydrophobic reduced GO-coated COC, and can control the behavior of surface wettability as needed. FTIR spectroscopy and contact angle measurements have confirmed the reduction of the GO coatings for each treatment. The results showed that HI acid reduction and thermal reduction increased the hydrophobicity of GO-coated COC more effectively than UV reduction. This can be attributed to the high removal of oxygen-containing groups in chemical and thermal reduction treatments compared to UV reduction. This work provides a basis for the further development of coating materials on COC substrates, where they can be used to tune the wettability for different industrial applications.

## Figures and Tables

**Table 1:** Surface roughness of plasma treated COC at different plasma exposure times.

Plasma Exposure Time (min)	Surface Roughness (nm)
0 (untreated COC)	15.7
5	33.4
20	43
120	122.3

## Data Availability

The data presented in this study are available in article.
